# Associations between clinical data, vaccination status, antibody responses, and post-COVID-19 symptoms in Thais infected with SARS-CoV-2 Delta and Omicron variants: a 1-year follow-up study

**DOI:** 10.1186/s12879-024-09999-2

**Published:** 2024-10-07

**Authors:** Wathusiri Khongsiri, Prapassorn Poolchanuan, Adul Dulsuk, Narin Thippornchai, Rungnapa Phunpang, Chakkaphan Runcharoen, Thitiya Boonprakob, Onura Hemtong, Suchada Chowplijit, Vachara Chuapaknam, Tanaya Siripoon, Watcharapong Piyaphanee, Viravarn Luvira, Chawarat Rotejanaprasert, Pornsawan Leaungwutiwong, Wasun Chantratita, Narisara Chantratita, Nathamon Kosoltanapiwat

**Affiliations:** 1 Department of Microbiology and Immunology, Faculty of Tropical Medicine, Mahidol University, Bangkok, 10400 Thailand; 2 Center for Medical Genomics, Faculty of Medicine Ramathibodi Hospital, Mahidol University, Bangkok, Thailand; 3 Prachatipat Hospital, Pathum Thani, Thailand; 4 Vichaivej International Hospital, Samut Sakhon, Thailand; 5 Department of Clinical Tropical Medicine, Faculty of Tropical Medicine, Mahidol University, Bangkok, Thailand; 6 Thai Travel Clinic, Hospital for Tropical Diseases, Faculty of Tropical Medicine, Mahidol University, Bangkok, Thailand; 7 Department of Tropical Hygiene, Faculty of Tropical Medicine, Mahidol University, Bangkok, Thailand; 8 Mahidol-Oxford Tropical Medicine Research Unit, Faculty of Tropical Medicine, Mahidol University, Bangkok, Thailand

**Keywords:** SARS-CoV-2, COVID-19, Delta, Omicron, Antibody response, ELISA, Thailand

## Abstract

**Background:**

Severe acute respiratory syndrome coronavirus 2 (SARS-CoV-2), which causes coronavirus disease 2019 (COVID-19), led to a global pandemic from 2020. In Thailand, five waves of outbreaks were recorded, with the fourth and fifth waves driven by the Delta and Omicron variants, resulting in over 20,000 new confirmed cases daily at their peaks.

**Methods:**

This cross-sectional study investigated the associations between clinical symptoms, vaccination status, antibody responses, and post-COVID-19 sequelae in COVID-19 patients. Plasma samples and clinical data were collected from participants admitted to hospitals in Thailand between July 2021 and August 2022, with follow-ups conducted for one year. The study included 110 participants infected with either the Delta (*n* = 46) or Omicron (*n* = 64) variants. Virus genotypes were confirmed by RT-PCR of nasal swab RNA and partial nucleotide sequencing of the S gene. IgG and IgA antibody levels against the receptor-binding domain (RBD) of SARS-CoV-2 Delta and Omicron variants were measured in plasma samples using ELISA.

**Results:**

Pneumonia was found to be associated with Delta variant infections, while sore throat, congestion or runny nose, and headache were linked to Omicron infections. Vaccination with fewer than two doses and diabetes mellitus were significantly associated with higher disease severity. Specific IgG and IgA antibodies against the RBD of the Delta variant generally rose by day 14 and were maintained for up to two months, whereas the pattern of antibody response to the Omicron variant was less clear. Antibody risings were found to be positively associated with pneumonia, certain underlying conditions (obesity, hypertension, dyslipidemia, and diabetes mellitus), and age ≥ 60 years. Delta variant infections were associated with forgetfulness, hair loss, and headache during the 1-year post-infection period. Females were more likely to experience hair loss, forgetfulness, and joint pain, while older age was associated with joint pain.

**Conclusions:**

This study enhances our understanding of SARS-CoV-2 infections in Thais, particularly concerning the Delta and Omicron variants. The findings can inform public health planning and response strategies for future outbreaks of SARS-CoV-2 or other emerging viral diseases.

**Supplementary Information:**

The online version contains supplementary material available at 10.1186/s12879-024-09999-2.

## Background

Coronavirus disease 2019 (COVID-19) is caused by severe acute respiratory syndrome coronavirus 2 (SARS-CoV-2), which belongs to the subgenus *Sarbecovirus* in the genus *Betacoronavirus* of the family Coronaviridae. SARS-CoV-2 is an enveloped virus with a positive-sense single-stranded RNA genome. The genome is almost 80% identical to that of SARS-CoV and 96.2% identical to the closest bat coronavirus (Bat-CoV RaTG13) [1, 2]. The virus has four major structural proteins, nucleocapsid (N), membrane (M), envelope (E), and spike (S), along with 16 non-structural proteins involved in transcription and virus replication [2]. The S protein, responsible for host cell attachment and virus entry, consists of the S1 and S2 subunits. The receptor-binding domain (RBD) is located in the S1 subunit and contains a receptor-binding motif (RBM) that facilitates the attachment of the virus to the human angiotensin-converting enzyme 2 (ACE2) receptor on the host cell. The RBD domain is a major target of neutralizing antibodies [2, 3]. SARS-CoV-2 infection typically causes flu-like symptoms such as fever, fatigue, and dry cough, although some cases are asymptomatic. More severe disease forms can also occur, leading to acute respiratory distress syndrome, cardiovascular complications, heart failure, hypoxemia, multiple organ dysfunction, or death, particularly in high-risk groups and patients with comorbid conditions [2, 4].

**Fig. 1:**
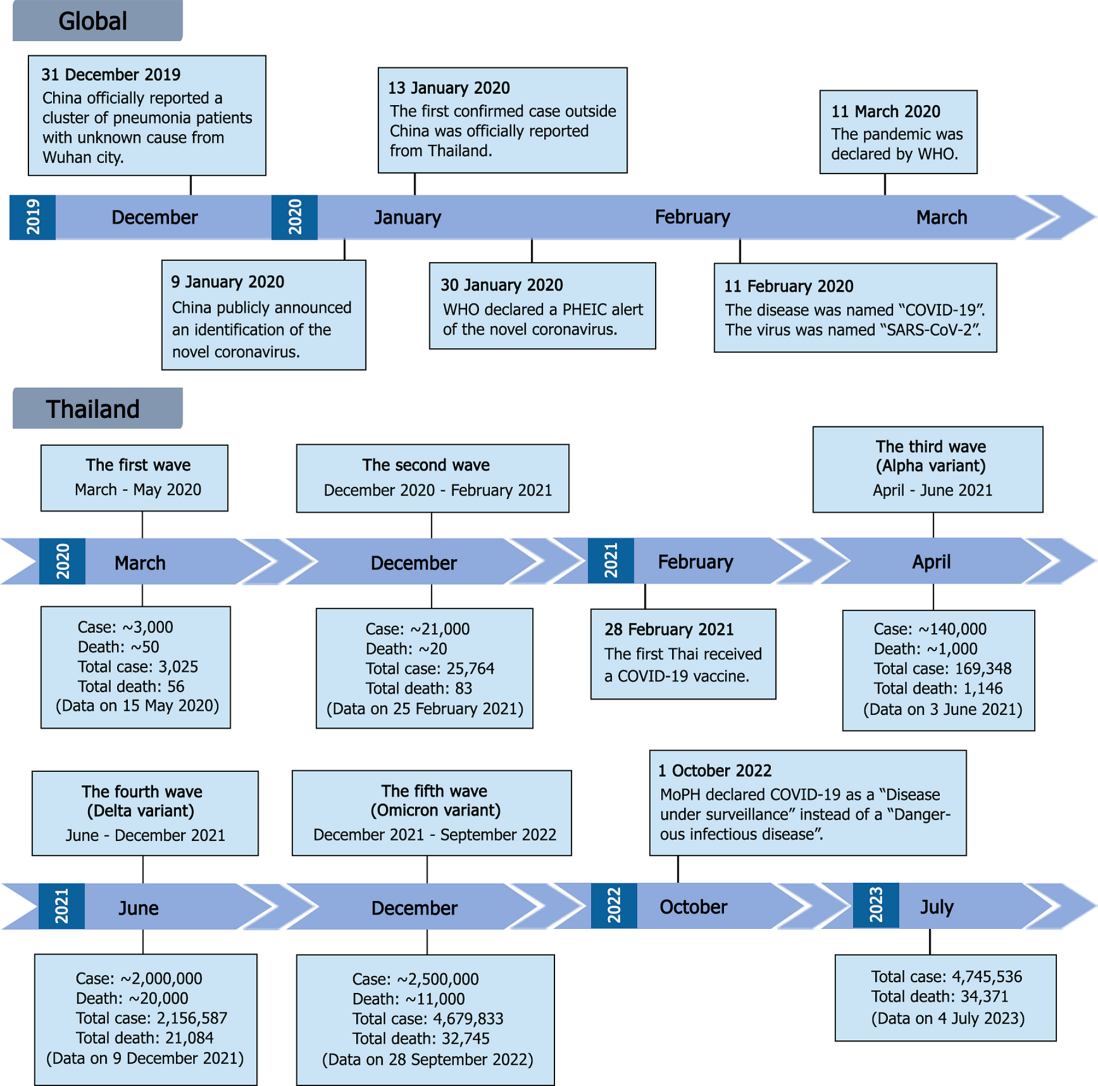
Timeline of COVID-19 pandemic progression and important events at global and national levels (Thailand). PHEIC, Public Health Emergency of International Concern; MoPH, Ministry of Public Health of Thailand; Case and Death, number of cases and deaths reported during a specific COVID-19 wave; Total case and Total death, cumulative cases and deaths reported by the Thai MoPH

The first SARS-CoV-2 infections were officially reported to the World Health Organization (WHO) on December 31, 2019, when a cluster of patients with pneumonia of an unknown cause emerged in Wuhan, China [2, 5]. In early January 2020, the etiologic virus was isolated from a patient, and its genome was sequenced and made publicly available [6]. After the novel coronavirus was identified, the first death was reported in China. Shortly thereafter, on January 13, 2020, the first confirmed case outside of China was officially reported in Thailand [5, 7]. Within a month, the novel coronavirus was found to be highly contagious and rapidly spreading, and on January 30, 2020, WHO declared it a public health emergency of international concern (PHEIC). On February 11, 2020, WHO named the disease caused by the novel coronavirus “COVID-19” short for “coronavirus disease 2019” [8], and the virus was named “SARS-CoV-2” by the International Committee on Taxonomy of Viruses (ICTV) [9]. A pandemic situation was officially declared by WHO on March 11, 2020, as the disease continued to spread worldwide, affecting at least 114 countries and resulting in more than 4,000 deaths [10] (Fig. 1).

In Thailand, five waves of COVID-19 outbreaks were reported between 2020 and 2022 [11]. The first wave, the smallest, occurred between March and May 2020, originating from gatherings at a boxing stadium and entertainment venues in Bangkok, with new daily cases peaking at over 100 [12, 13]. By the end of this wave, Thailand had reported approximately 3,000 confirmed cases and around 50 deaths. The disease appeared to be well-controlled by strong public health and social measures until December 2020, when an outbreak was detected at a shrimp market in Samut Sakhon Province [14, 15]. This second wave resulted in further outbreaks across the country, with local transmission chains clearly identified and with more than 800 daily new cases at the peak. Containment was eventually achieved by February 2021. By the end of February, there had been approximately 26,000 confirmed cases and around 80 deaths, with about 20,000 cases reported during the second wave. It was at this time that COVID-19 vaccines began to be distributed in Thailand [16]. The first used in Thailand was an inactivated vaccine produced from the wild-type Wuhan strain.

The third wave, which began in April 2021, was predominantly caused by the Alpha variant of SARS-CoV-2 [11] and was followed by a fourth wave in June 2021 driven by the Delta variant [15, 17]. The fifth wave, starting in December 2021, was marked by the emergence of the Omicron variant [18]. The Delta and Omicron waves saw the highest numbers of accumulated cases and deaths. Despite vaccination efforts, with 55 per 1,000 population receiving the first dose and 20 per 1,000 population receiving the second dose by June 2021, new confirmed cases peaked at over 20,000 and 25,000 per day during the Delta and Omicron waves, respectively [17]. By the end of September 2022, Thailand had reported approximately 4.7 million confirmed cases and more than 30,000 deaths, with the majority of these occurring during the Delta and Omicron outbreaks (Fig. 1).

There is a study from Thailand that describes the molecular characterization and tracking of SARS-CoV-2 variants from the first to the fifth epidemic wave [19]. The study emphasized the importance of continued molecular surveillance of SARS-CoV-2 for monitoring new variants that could potentially cause future outbreaks. However, there has been a lack of studies aiming to understand how different SARS-CoV-2 variants affect immune responses, clinical outcomes, and post-COVID-19 outcomes in Thailand. Therefore, samples and clinical data were collected from COVID-19 patients admitted between July 2021 and August 2022 to three hospitals in Thailand. During this period, the Delta and Omicron variants were the dominant circulating strains. Sequential plasma samples were collected along with clinical data from the date of admission until 1-year post-infection. Levels of plasma antibodies against the SARS-CoV-2 Delta and Omicron variants were determined using a SARS-CoV-2 RBD-specific ELISA. Virus genotypes were confirmed by reverse-transcription (RT)-PCR of nasal swab samples and partial nucleotide sequencing of the S gene. The data were analyzed to determine associations between demographic factors, clinical characteristics, vaccination status, antibody responses, and post-COVID-19 outcomes, with a focus on comparative analysis of Delta- and Omicron-infected groups. Although we are now adjusting to living with COVID-19, the information gained from this descriptive research can provide a better understanding of SARS-CoV-2, particularly in the context of Delta and Omicron infections, and offer valuable lessons on how to prepare for future pandemics.

## Methods

### Study sites and samples

Samples and data were collected from 110 COVID-19-confirmed cases between 2021 and 2023. Participant enrollment, as well as sample and data collection, began in July 2021 and continued until August 2022 (day 0). The participants were followed for 1 year (until August 2023). Subjects were admitted to the Hospital for Tropical Diseases, Bangkok (Hospital A, *n* = 87), Vichaivej International Hospital, Samut Sakhon (Hospital B, *n* = 7), and Prachathipat Hospital, Pathum Thani (Hospital C, *n* = 16), Thailand. A total of 330 plasma samples were obtained at different time points. Acute plasma samples were collected within the first week of disease confirmation (≤ 7 days), defined as day 0. Convalescent plasma samples were collected on day 14, day 28 and at 1 year at Hospital A, and on day 14, month 2, month 6, and at 1 year at Hospitals B and C (Table 1). Viral RNA extracted from nasal swabs of patients enrolled at Hospital A (*n* = 52) was obtained for virus variant identification. Questionnaire was used to collect the participants’ post-COVID-19 information at day 28 and year 1 after enrollment (Supplementary file).

**Table 1:** Numbers and dates of plasma sample collections

Date of collection	Hospital A	Hospital B	Hospital C	Total
Day 0	87	6	16	109
Day 14	43	7	15	65
Day 28	65	NA	NA	65
Month 2	NA	7	16	23
Month 6	NA	5	15	20
Year 1	42	0	6	48
**Total**	237	25	68	330

NA, Samples were not collected at the time points

### RT-PCR and nucleotide sequencing of SARS-CoV-2 partial spike gene

RT-PCR was performed to amplify a partial spike gene of SARS-CoV-2 from RNA extracted from nasal swabs using the SuperScript™ III One-Step RT-PCR System with Platinum™ *Taq* DNA Polymerase. The RT-PCR reaction mixture was prepared according to the manufacturer’s instructions, containing 0.2 µM of each forward and reverse primer and 5 µL of RNA in a total volume of 50 µL. The primers SP1sense (5′-GTTTGTTTTTCTTGTTTTATT-3′) and ASP1antisense (5′-ACAGTGAAGGATTTCAACGTACAC-3′) [20] were designed to bind to a region spanning the N-terminal domain (NTD) and receptor-binding domain (RBD) of the S1 subunit of the SARS-CoV-2 spike gene (position 21,565 − 22,485, reference genome accession no. NC_ 045512-Wuhan-HU-1). The RT-PCR conditions were as follows: 60 °C for 1 min, 50 °C for 45 min, and 94 °C for 2 min, followed by 40 cycles of 95 °C for 15 s, 53 °C for 30 s and 68 °C for 1 min, with a final extension at 68 °C for 7 min. PCR products of approximately 900 bp were observed by gel electrophoresis, purified and sent for Sanger sequencing using the amplification primers. The retrieved nucleotide sequences were analyzed by BioEdit V7.2.5 and queried against available published SARS-CoV-2 sequences in the GenBank database. A phylogenetic tree was constructed using the Molecular Evolutionary Genetics Analysis (MEGA, version 7.0.26) software, employing the maximum likelihood method based on the Tamura 3-parameter model. SARS-CoV-2 partial S gene sequences (approximately 870 bp) from 52 samples collected at Hospital A were submitted to the GenBank database and received accession numbers PP411428 to PP411479. The reference sequences included NC_045512.2 (Wuhan-Hu-1), OK084567.1 (Alpha variant), OK084639.1 (Delta variant), OM984777.1 (Omicron BA.1 variant), OM984826.1 (Omicron BA.2 variant), and MN996532.2 (Bat coronavirus RaTG13).

### Identification of SARS-CoV-2 variants by MassARRAY® system

MassARRAY^®^ was used for the identification of SARS-CoV-2 variants in samples from Hospitals B and C as previously described [21]. Viral RNA extracted from participants’ respiratory samples was converted to cDNA using the SuperScript™ IV First-Strand Synthesis System (Invitrogen) with random hexamers. The cDNA was then subjected to PCR multiplex reactions performed in three steps according to the manufacturer’s protocol. Briefly, in step 1, multiplex PCR was performed using a hot start *Taq* polymerase (Agena Bioscience) with SARS-CoV-2 specific amplification primers [21]. In step 2, the PCR products from step 1 were treated with shrimp alkaline phosphatase (Agena Bioscience) to inhibit the functioning of amplification primers and dNTPs. In step 3, a single base extension reaction was carried out using the mixture from step 2 with iPlex enzyme (Agena Bioscience) and extension primers [21]. The step 3 solutions were desalted using ion exchange resin (Agena Bioscience), spotted onto a matrix pre-coated 96-SpectroCHIP^®^ Array (Agena Bioscience), and subjected to mass spectrometry assay using a MassARRAY^®^ MALDI-TOF MS system (Agena Bioscience) according to the manufacturer’s instructions. SARS-CoV-2 genotypes were analyzed using a MassARRAY^®^ Typer v4.0 (Agena Bioscience).

### SARS-CoV-2 RBD-specific ELISA

IgG and IgA antibodies in plasma were detected using an enzyme-linked immunosorbent assay (ELISA) specific to the receptor-binding domain (RBD) proteins of SARS-CoV-2 variants, Delta and Omicron, as previously described [22]. An ELISA plate (Nunc MaxiSorp U-bottom 96-Well plate) was coated with recombinant RBD antigens (GenScript, Z03613 for the Delta variant; Z03740-1 for the Omicron variant). The RBD antigen concentrations were 2 µg/mL for IgG and 4 µg/mL for IgA detection. Fifty microliters of RBD antigen in 0.05 M sodium carbonate buffer (pH 9.6) were coated and incubated overnight at 4°C. Sample wells were washed four times with 300 µL of phosphate-buffered saline (PBS) containing 0.05% Tween-20 (PBST) using a TECAN Hydrospeed washer and blocked with 200 µL of 5% skim milk in PBS at 37°C for 2 hours. After removing the blocking solution, fifty microliters of 1:100 diluted plasma samples, positive controls, or negative controls (in PBST containing 1% bovine serum albumin) were added to antigen-coated and uncoated wells and incubated in a moist chamber at room temperature for 1 hour. Wells were then washed as described above. Fifty microliters of horseradish peroxidase (HRP)-conjugated anti-human immunoglobulins were added into each well and incubated at room temperature for 1 hour. HRP-conjugated anti-human IgA (Invitrogen) and anti-human IgG (DAKO) were used at dilutions of 1:2000 and 1:4000, respectively. Wells were washed, and 50 µL of 3,3’,5,5’-tetramethylbenzidine (TMB) substrate (Novex, Life Technologies) was added for color development. The reaction was stopped after incubating at room temperature for 15 min by the addition of 50 µL 1 N HCl. The absorbance was measured at an optical density (OD) of 450 nm with a Sunrise^™^ microplate reader (TECAN). Pooled plasma samples from COVID-19 patients (*n* = 10) were used as a positive control, and pooled pre-vaccination donor plasma samples (*n* = 10) were used as a negative control. The OD value of a blank, which contained only the assay diluent (PBST with bovine serum albumin), was subtracted from all the OD values of test samples. The OD values of individual samples in uncoated wells were measured. Antibody levels were determined by subtracting the OD value in uncoated wells from the OD value in antigen-coated wells. An OD value of 0.01 was interpreted as 1 unit (U)/mL of antibody.

### Statistical analysis

Analysis of associations between categorical data (age, sex, infected variant, severity, vaccination status, clinical symptoms, underlying conditions, post-COVID-19 sequelae, and antibody ratio) was performed using Pearson’s chi-square test. Common odds ratios were estimated using the Mantel-Haenszel method, and adjusted odds ratios were estimated using logistic regression analysis in PASW Statistics 18. Medians of antibody levels were compared using the Mann-Whitney U test in GraphPad Prism version 9.0. *p* values < 0.05 were considered statistically significant.

### Ethics approval and consenting

The use of samples and data in this study was approved by the Ethics Committee of the Faculty of Tropical Medicine, Mahidol University (Approval No. MUTM 2023-048-01). The human samples used in this work were leftover from previous EC-approved studies (Approval No. MUTM 2021-028-03 and MURA2021/264), for which informed consent had been obtained. During the consent process for those studies, participants provided permission to use their leftover specimens and data for further research.

## Results

### Participant information

Participant demographic data and infected SARS-CoV-2 variants are shown in Table 2. The median age of all participants was 53 years (range 18–89 years). The median age of participants from Hospital A was 56 years (range 18–89 years), while that of participants from Hospitals B and C combined was 42 years (range 19–62 years). Because a small number of patients were enrolled from Hospitals B and C and the same time point was used for sample collection, data from these two hospitals were combined. Among all participants, 40.9% were male and 59.1% were female; 41.8% were infected with the Delta variant and 58.2% with the Omicron variant. When participants from Hospital A were grouped by variant infection, the median ages were 50 years (range 18–72 years) for the Delta-infected group and 59 years (range 26–89 years) for the Omicron-infected group. The numbers of male and female participants were equal in the Delta-infected group, while the Omicron-infected group had more female than male participants, at a ratio of 1:1.7. No associations among age, gender, or infected variant were detected by chi-square test.

**Table 2:** Participant demographic data and SARS-CoV-2 variant infection

All Participants		Hospital A (*n* = 87)	Hospital B (*n* = 7)	Hospital C (*n* = 16)	Total (*n* = 110)
Age	18–59	56 (64.4%)	7 (100.0%)	13 (81.3%)	76 (69.1%)
	≥ 60	31 (35.6%)	0	3 (18.7%)	34 (30.9%)
Gender	Male	38 (43.7%)	1 (14.3%)	6 (37.5%)	45 (40.9%)
	Female	49 (56.3%)	6 (85.7%)	10 (62.5%)	65 (59.1%)
SARS-CoV-2	Delta	46 (52.9%)	0	0	46 (41.8%)
	Omicron	41 (47.1%)	7 (100%)	16 (100.0%)	64 (58.2%)
**Participants from Hospital A infected with**		**Delta** **(** ***n*** **= 46)**	**Omicron** **(** ***n*** **= 41)**		**Total** **(** ***n*** **= 87)**
Age	18–59	33 (71.7%)	23 (56.1%)		56 (64.4%)
	≥ 60	13 (28.3%)	18 (43.9%)		31 (35.6%)
Gender	Male	23 (50.0%)	15 (36.6%)		38 (43.7%)
	Female	23 (50.0%)	26 (63.4%)		49 (56.3%)

### Detection of SARS-CoV-2 variant distribution

**Fig. 2:**
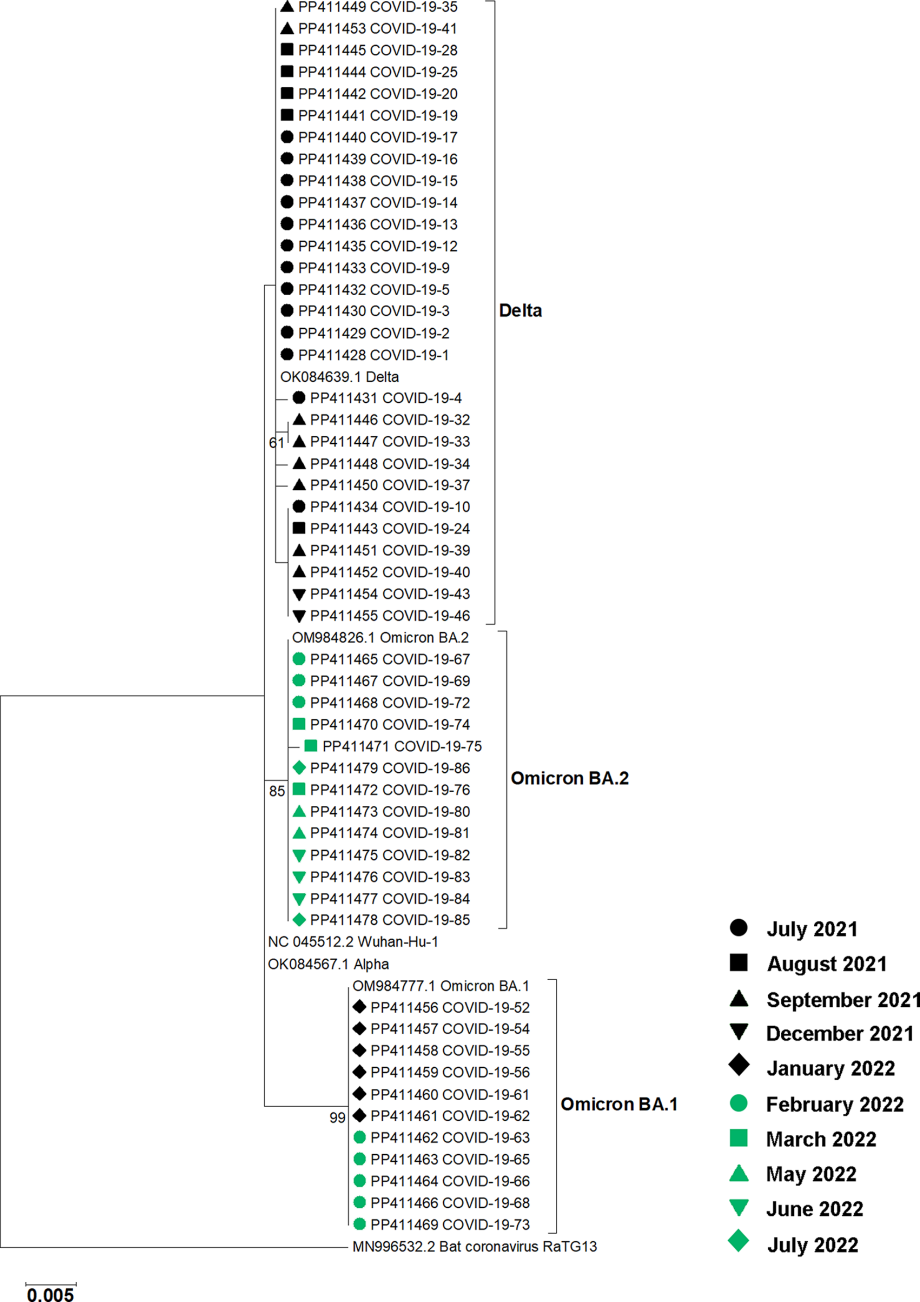
Phylogenetic analysis of SARS-CoV-2 partial S gene sequences from 52 nasal swab samples collected at Hospital A. The tree was constructed using the maximum likelihood method with a bootstrap value of 1,000. Accession numbers for sequences obtained in this study and reference sequences are shown. Labels indicating the months of sample collection are shown. Bootstrap values greater than 50 are indicated at the nodes. The bar represents nucleotide substitutions per site. A bat coronavirus sequence was used as an outgroup

RT-PCR of the partial SARS-CoV-2 spike gene and nucleotide sequencing were used to determine virus variants present in nasal swabs from participants at Hospital A. RNA from nasal swabs was available for molecular genotyping for 52 of the 87 participants. Phylogenetic analysis revealed that 28 samples collected between July and December 2021 contained the Delta variant, while 24 samples collected between January and August 2022 contained the Omicron variant. Among the Omicron sequences, 11 were identified as the BA.1 subvariant, and 13 were classified as the BA.2 subvariant (Fig. 2). Virus variant genotyping for samples from Hospitals B and C was performed elsewhere using MassARRAY^®^ system [21]. All samples from Hospitals B and C, collected during January and August 2022, contained the Omicron variant. For the 35 participants from Hospital A with no genotyping results available, virus variants were assumed based on the time of sample collection. Because the first Omicron case was reported in Thailand on December 8, 2021 [23], untyped samples collected before that date were assigned to the Delta variant, while those collected after were assigned to the Omicron variant. Overall, 46 participants were infected with the Delta variant, and 64 patients were infected with the Omicron variant (Total *n* = 110) (Table 2). The Delta variant was detected in this study until December 2021, while the Omicron variant was detected from December 2021. From January 2022 onward, only the Omicron variant was detected.

### Analysis of participants’ clinical presentations, underlying conditions, and vaccination history at enrollment

**Fig. 3:**
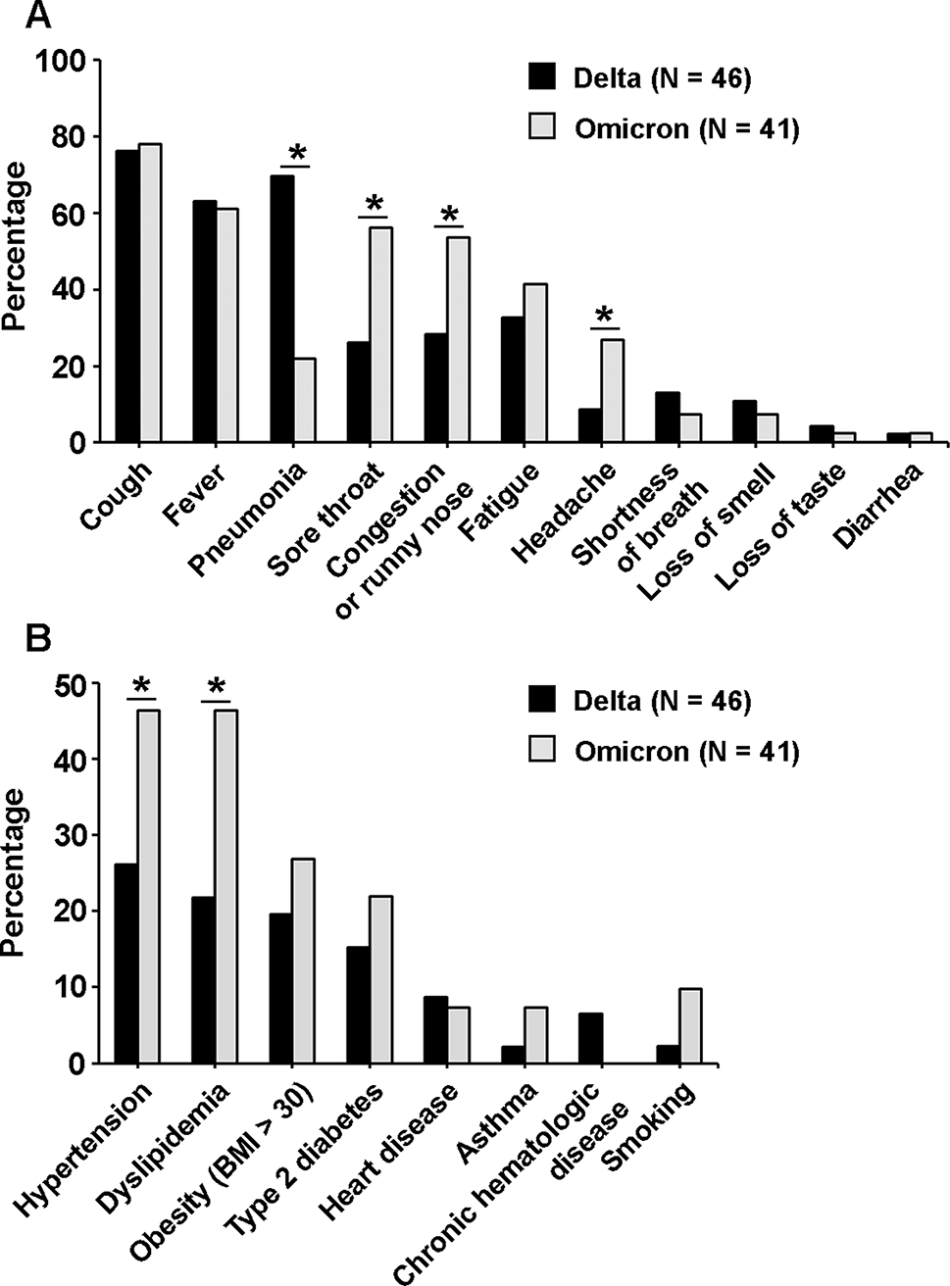
Clinical presentations and underlying conditions of participants infected with SARS-CoV-2 Delta or Omicron variants from Hospital A. The percentage of participants presenting with different clinical symptoms (**A**) and underlying conditions (**B**) were compared between those infected with the Delta variant (black bars, *n* = 46) and the Omicron variant (grey bars, *n* = 41). A chi-square test was used to identify clinical presentations or underlying conditions associated with infection by a particular SARS-CoV-2 variant. Stars indicate statistical significance at *p* ≤ 0.05

Clinical presentations and underlying conditions of the participants from Hospital A (*n* = 87) were collected on the date of enrollment. Regardless of the infecting variant, the clinical presentations reported, in descending order of frequency, were cough (77.0%), fever (62.1%), pneumonia (47.1%), sore throat (40.2%), congestion or runny nose (40.2%), fatigue (36.8%), headache (17.2%), shortness of breath (10.3%), loss of smell (anosmia) (9.2%), loss of taste (ageusia) (3.4%), and diarrhea (2.3%). Cough (76.1% and 78.0%) and fever (63.0% and 61.0%) were generally observed in participants infected with either the Delta or Omicron variants, respectively. Pneumonia was found in the majority of participants infected with the Delta variant (69.6%) and less of those with the Omicron variant (22.0%), whereas sore throat (56.1% vs. 26.1%), congestion or runny nose (53.7% vs. 28.3%), and headache (26.8% vs. 8.7%) were more prominent in participants infected with the Omicron variant than the Delta variant, with a *p* ≤ 0.05 (Fig. 3A). A risk estimation of clinical presentations significantly associated with a particular SARS-CoV-2 variant infection is demonstrated by the odds ratios in Table 3. As other factors, such as age, sex, and underlying conditions, may be associated with clinical outcomes, these were also analyzed. No associations with age, sex, or underlying conditions were found for pneumonia, sore throat, congestion or runny nose, or headache. However, it was found that asthma and chronic hematologic disease were underlying conditions associated with an increased chance of shortness of breath during SARS-CoV-2 infection (Table 3).

**Table 3:** Factors associated with clinical presentations and underlying conditions

Presentations/conditions	Factors	Percentage^a^	χ^2^ *p*-value	Odds ratio	95% CI	*p*-value
**Clinical presentations**						
Pneumonia	Delta	69.6%	<0.001*	8.127	3.080–21.443	<0.001*
	Omicron	22.0%				
Sore throat	Delta	26.1%	0.004*	0.276	0.112–0.681	0.005*
	Omicron	56.1%				
Congestion or runny nose	Delta	28.3%	0.016*	0.340	0.140–0.827	0.017*
	Omicron	53.7%				
Headache	Delta	8.7%	0.025*	0.260	0.075–0.894	0.033*
	Omicron	26.8%				
Shortness of breath	Asthma	50.0%	0.008*	10.857	1.320–89.312	0.027*
	No Asthma	8.4%				
	Hematologic disease	66.7%	0.001*	22.000	1.766–273.997	0.016*
	No hematologic disease	8.3%				
**Underlying conditions**						
Hypertension	Delta	26.1%	0.049*	0.409	0.166–1.005	0.051
	Omicron	46.3%				
	Age ≥ 60	64.5%	<0.001*	7.438	2.770–19.975	<0.001*
	Age 18–59	19.6%				
Dyslipidemia	Delta	21.7%	0.015*	0.322	0.127–0.816	0.017*
	Omicron	46.3%				
	Age ≥ 60	61.3%	<0.001*	7.283	2.693–19.699	<0.001*
	Age 18–59	17.9%				
Obesity	Age ≥ 60	9.7%	0.028*	0.246	0.066–0.920	0.037*
	Age 18–59	30.4%				
Diabetes	Age ≥ 60	32.3%	0.013*	3.968	1.278–12.324	0.017*
	Age 18–59	10.7%				
**Adjusted odds ratio**						
Dyslipidemia	Delta			0.361	0.130–1.005	0.051
	Omicron					
	Age ≥ 60			6.819	2.456–18.931	<0.001*
	Age 18–59					

a, Percentage of participants presented with the clinical presentations or conditions in each factor category; *, statistically significant at *p* ≤ 0.05

For all participants, regardless of the infecting variant, hypertension was the most prevalent underlying condition (35.6%), followed by dyslipidemia (33.3%), obesity (23.0%), and type 2 diabetes mellitus (18.4%) (Fig. 3B). Heart disease (8.0%), asthma (4.6%), and chronic hematologic disease (3.4%) were reported in a smaller proportion of participants. Smoking was also recorded among the participants. Only a small proportion reported currently smoking (5.7%). Hypertension and dyslipidemia were more prevalent among Omicron-infected participants (46.3%) than Delta-infected participants (26.1% and 21.7%, respectively), with a *p* ≤ 0.05. By odds ratio estimation, only dyslipidemia was found to be associated with Omicron infection at *p* ≤ 0.05 (Table 3). Associations between age and sex with underlying conditions were also determined. No association between sex and underlying conditions was observed. However, an age of ≥ 60 years was found to be associated with hypertension, dyslipidemia, and diabetes mellitus, whereas a younger age (18–59 years) was associated with obesity in the tested population. Because both virus variant and age were significantly associated with dyslipidemia, the adjusted odds ratios for these factors were calculated (Table 3). In general, while participants’ ages were associated with some reported underlying conditions, infections with different SARS-CoV-2 variants were not.

**Fig. 4:**
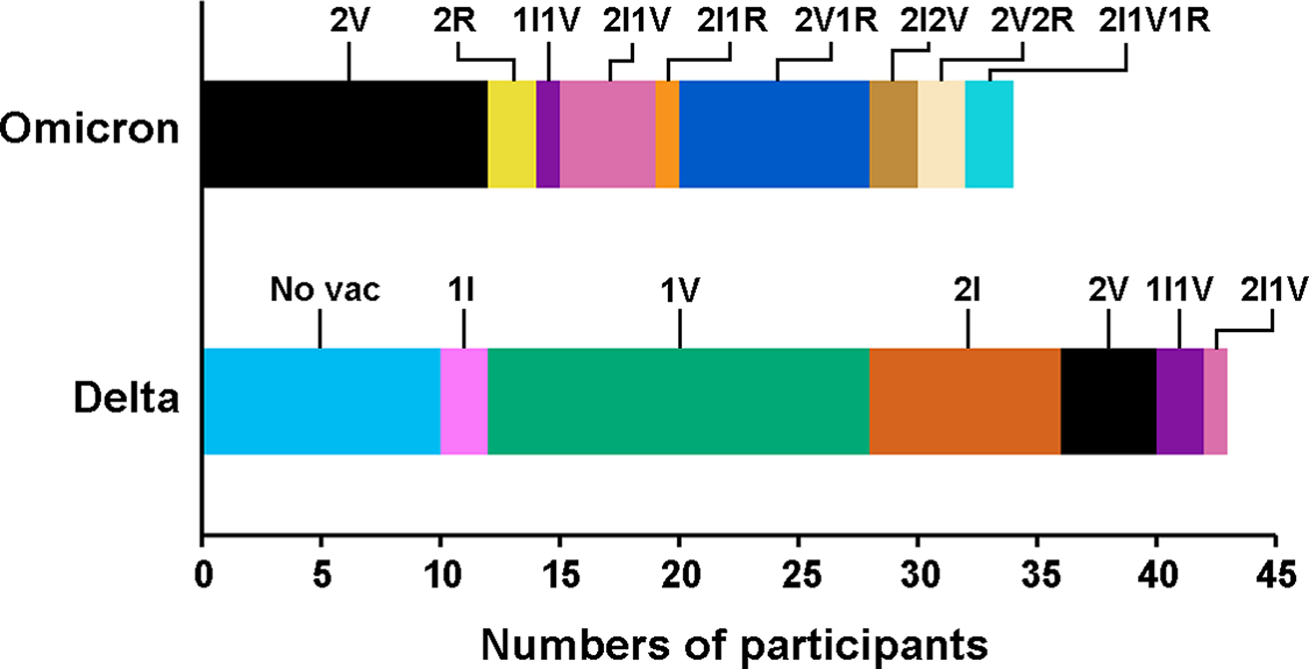
COVID-19 vaccination history of participants from Hospital A prior to SARS-CoV-2 infection. Vaccination history was available for 77 participants, with 43 infected by the Delta variant and 34 by the Omicron variant. Different vaccine combinations are represented by color labels: No vac, no history of COVID-19 vaccination; I, inactivated vaccine; V, viral vector vaccine; R, mRNA vaccine

Vaccination history was available for 77 participants from Hospital A (Delta infection *n* = 43, Omicron infection *n* = 34). Among participants infected with the Delta variant, 10 (23.3%) were unvaccinated, while 18 (41.9%), 14 (32.6%), and 1 (2.3%) had received 1, 2, and 3 doses, respectively, of various combinations of inactivated, viral vector-based, or mRNA COVID-19 vaccines (Fig. 4). All participants infected with the Omicron variant were vaccinated, with 15 (44.1%), 13 (38.2%), and 6 (17.6%) having received 2, 3, and 4 doses of vaccine, respectively. The association between vaccination and disease severity was tested. It was noted that only a few participants experienced life-threatening infections; specifically, 3 out of 87 participants required ICU admission, and none required mechanical ventilation. Therefore, the disease severity criteria used in this study were: (1) the participant had an oxygen saturation level (SpO_2_) less than 94% at admission [24], and (2) the participant received supplemental O_2_ during admission until discharge. If either criterion was met, the participant was defined as severe. Vaccination with one shot or less was found to be associated with an increased chance of severity (Table 4). Age, sex, infection variant, underlying conditions, and smoking were included as other factors that may have contributed to disease severity. Although age, gender, SARS-CoV-2 variant, and smoking were not associated with disease severity, diabetes mellitus did show an association. The adjusted odds ratios for vaccination and diabetes factors were determined. Collectively, vaccination with one shot or less and diabetes mellitus were found to be significantly associated with disease severity (Table 4). In addition, an association between non-booster (1 dose) and booster (more than 1 dose) vaccination with disease severity was analyzed. The result suggested that non-booster status seemed to be associated with an increased chance of disease severity (odds ratio 2.563, 95% CI 0.743–8.843), but this was not statistically significant (*p* = 0.136).

**Table 4:** Vaccination status and underlying condition associated with disease severity

Factors	Percentage^a^	χ^2^ *p*-value	Odds ratio	95% CI	*p*-value
Vaccinated with ≤ 1 dose	42.9%	0.011*	3.844	1.325–11.149	0.013*
Vaccinated with > 1 dose	16.3%				
Diabetes	50.0%	0.007*	4.462	1.413–14.088	0.011*
No diabetes	18.3%				
**Adjusted odds ratio**					
Vaccinated with ≤ 1 dose			5.021	1.540–16.368	0.007*
Vaccinated with > 1 dose					
Diabetes			5.613	1.523–20.684	0.010*
No diabetes					

a, Percentage of participants presented with severity in each factor category; *, statistically significant at *p* ≤ 0.05

### SARS-CoV-2 RBD-specific IgG and IgA responses

**Fig. 5:**
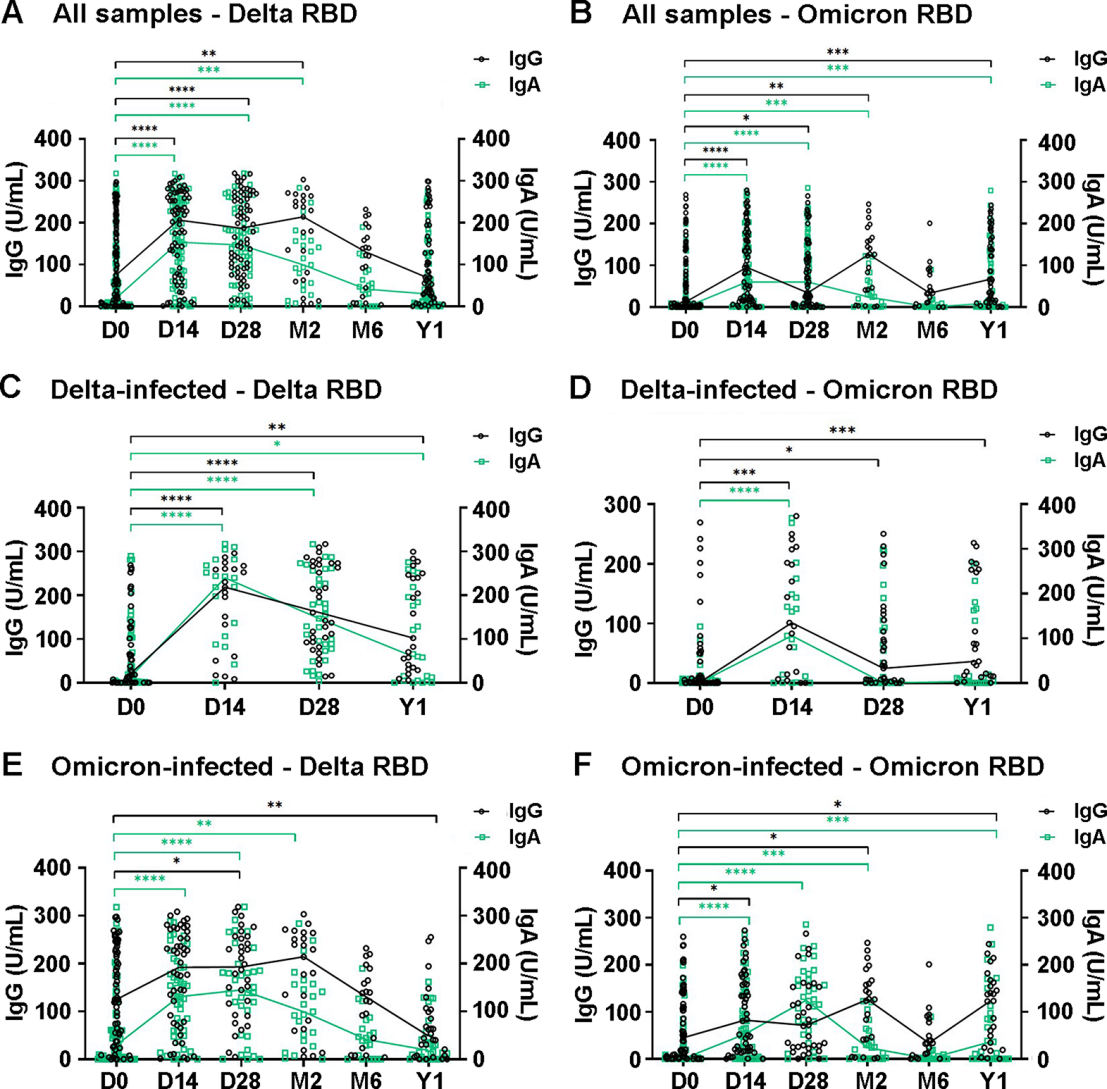
Levels of RBD-specific IgG and IgA antibodies in plasma over a one-year follow-up period. IgG levels are represented on the left y-axis, and IgA levels on the right y-axis. (**A**) and (**B**) show antibody levels to the RBD of Delta and Omicron variants, respectively, in all samples. (**C**) and (**D**) show antibody levels in Delta-infected samples from Hospital A. (**E**) and (**F**) show antibody levels in Omicron-infected samples from Hospitals A, B, and C. The Mann-Whitney U test was used to compare the medians of non-normally distributed data. *, *p* ≤ 0.05; **, *p* ≤ 0.01; ***, *p* ≤ 0.001; and ****, *p* ≤ 0.0001 indicate statistical significance

Levels of IgG and IgA antibodies against the RBD of the SARS-CoV-2 S protein in plasma samples of COVID-19 patients were determined by ELISA (Fig. 5). Overall IgG and IgA levels against Delta and Omicron RBD antigens were shown for all samples collected on days 0, 14, 28; months 2 and 6; and 1 year from all sites, irrespective of the infected variants. IgG and IgA levels against the Delta RBD peaked on day 14 and were maintained until around month 2, but then declined (Fig. 5A). In month 6, the levels were comparable to those on day 0. In contrast, IgG and IgA levels against the Omicron-RBD were elevated on day 14 and thereafter, but the pattern of antibody increase was not as clear as that observed for antibodies against the Delta RBD (Fig. 5B). When samples were analyzed separately according to the infecting variants, a rising in IgG and IgA levels against the Delta RBD was observed in the Delta-infected group on day 14 and remained detectable for 1 year, although with a declining trend (Fig. 5C). Cross-reactive IgG and IgA against the Omicron RBD were observed in the Delta-infected group on day 14 (Fig. 5D). Cross-reactive IgG and/or IgA against the Delta RBD were also observed in the Omicron-infected group on days 14 and 28 and month 2 (Fig. 5E). Notably, in the Omicron-infected group, the increase in cross-reactive IgG and IgA levels against the Delta-RBD was more pronounced than the increase in antibodies against the Omicron RBD (Fig. 5F).

### Factors associated with antibody responses in Delta- and omicron-infected participants

Associations between antibody levels and factors such as age, sex, severity, clinical symptoms (fever and pneumonia), and underlying conditions (hypertension, dyslipidemia, obesity, diabetes mellitus, heart disease, asthma, and chronic hematologic disease) were determined in the Delta- and Omicron-infected groups of participants from Hospital A. To categorize antibody levels, the ratios of antibody levels on day 0 to those on convalescent days were calculated. A rise in antibody levels was defined as a < 2-fold or ≥ 2-fold increase. Age, sex, and severity were not associated with antibody responses in this study. In the Delta-infected group, pneumonia was found to be associated with a ≥ 2-fold increase in Delta-specific antibodies, whereas obesity, hypertension, and dyslipidemia were associated with a ≥ 2-fold increase in cross-reactive Omicron antibodies (Table 5). Obesity was also observed to be associated with a decrease in prolonged Delta IgG response in the Delta-infected group at year 1. In the Omicron-infected group, hypertension and diabetes were associated with a ≥ 2-fold increase in Omicron-specific IgA, whereas advanced age (≥ 60 years) was associated with an increase in the levels of cross-reactive IgG against the Delta variant.

**Table 5:** Factors associated with antibody responses in Delta- and omicron-infected groups of participants at Hospital A

Ab rising ≥ 2-fold compared to day 0	Factors	Percentage^a^	χ^2^ *p*-value	Odds ratio	95% CI	*p*-value
**Delta-infected group**						
Delta IgG at day 14	Pneumonia	85.7%	0.007*	24.000	1.689–340.992	0.019*
	No pneumonia	20.0%				
Delta IgG at year 1	Obesity	25.0%	0.014*	0.063	0.005–0.823	0.035*
	Normal BMI	84.2%				
Delta IgA at day 28	Pneumonia	77.3%	0.021*	5.950	1.223–28.951	0.027*
	No pneumonia	36.4%				
Omicron IgG at day 28	Obesity	75.0%	0.032*	6.375	1.046–38.858	0.045*
	Normal BMI	32.0%				
Omicron IgA at day 14	Hypertension	88.9%	0.027*	12.000	1.053–136.794	0.045*
	No hypertension	40.0%				
	Dyslipidemia	100.0%	0.047*	-	-	-
	No dyslipidemia	50.0%				
**Omicron-infected group**						
Delta IgG at day 14	Age ≥ 60	55.6%	0.028*	8.125	1.115–59.212	0.039*
	Age 18–59	13.3%				
Omicron IgA at day 14	Hypertension	100.0%	0.028*	-	-	-
	No hypertension	60.0%				
Omicron IgA at day 28	Diabetes	100.0%	0.028*	-	-	-
	No diabetes	58.3%				

a, Percentage of participants presented with antibody rising ≥ 2-fold in each factor category; *, statistically significant at *p* ≤ 0.05

### Participants’ post-COVID-19 sequelae

Follow-up information on post-COVID-19 sequelae was collected from participants at Hospital A 28 days (total, *n* = 87; Delta, *n* = 46; Omicron, *n* = 41) and 1 year (total, *n* = 70; Delta, *n* = 34; Omicron *n* = 36) post-infection. At day 28 post-infection (pi), regardless of the infected variant, the most reported symptoms were fatigue (29.9%) and cough (25.3%), followed by shortness of breath (18.4%) and forgetfulness (6.9%). Other reported symptoms (hair loss, chest pain, joint pain, headache, difficulty with thinking, and muscle pain) were reported by less than 5% of participants. At 1 year pi, irrespective of the infected variant, the most reported symptoms were hair loss (22.9%), followed by forgetfulness (17.1%), fatigue (12.9%), muscle pain (11.4%), cough (10.0%), joint pain (8.6%), shortness of breath and chest pain (7.1%), headache (5.7%), and difficulty with thinking (4.3%).

**Fig. 6:**
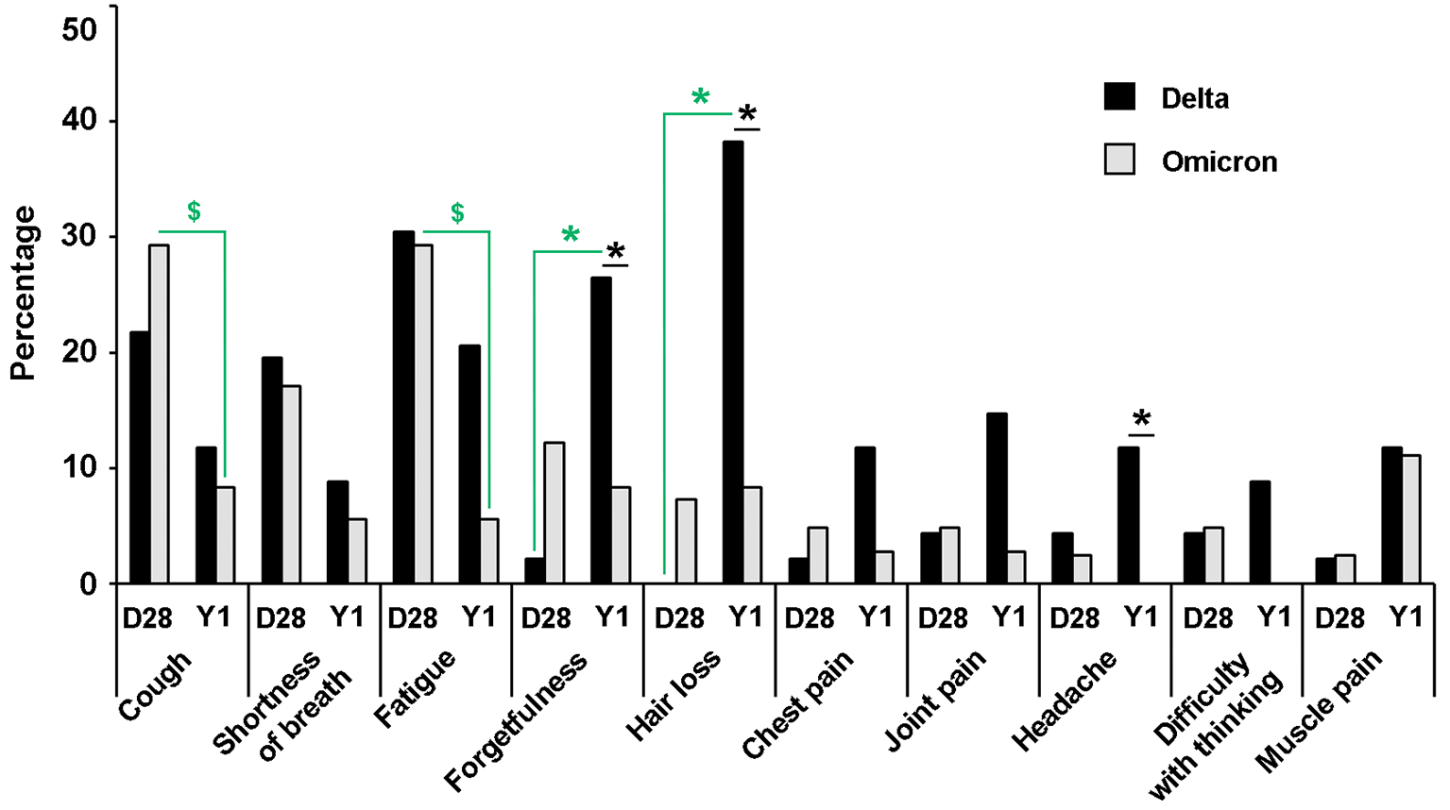
Post-COVID-19 sequelae reported at day 28 and one year after infection. The percentage of participants reporting different sequelae is shown for groups infected with the Delta variant (black bars) and the Omicron variant (grey bars). Pearson’s chi-square test was used to compare the number of participants reporting post-COVID-19 sequelae between groups infected with different variants and at different time points post-infection. Stars and dollar signs indicate statistical significance at *p* ≤ 0.05. Green stars represent significant differences in Delta-infected participants between day 28 and year 1. Green dollar signs represent significant differences in Omicron-infected participants between day 28 and year 1. Black stars represent significant differences between Delta and Omicron infections

The cough, shortness of breath, and fatigue reported on day 28 seemed to resolve within 1 year in both Delta- and Omicron-infected groups (Fig. 6). The cough and fatigue reported by Omicron-infected participants on day 28 pi (29.3%) significantly decreased by 1 year pi (8.3% and 5.6%, respectively, *p* ≤ 0.05) (Fig. 6; Table 6). In contrast, forgetfulness and hair loss were less likely to be noticed on day 28 pi (< 3%) but were significantly more frequently reported at 1 year pi in the Delta-infected group (26.5% and 38.2%, respectively, *p* ≤ 0.05). At 1 year pi, forgetfulness, hair loss, and headache were significantly more prevalent among participants infected with the Delta variant than those infected with the Omicron variant (*p* ≤ 0.05, chi-square). However, by odds ratio estimation, statistical significance was found only for hair loss with the Delta infection (Table 6). Age and sex were included in the analysis as factors that may affect post-COVID-19 sequelae. Older adults (age ≥ 60) reported joint pain on day 28 pi more frequently than younger participants. Females were more likely than males to report forgetfulness on day 28 and hair loss and joint pain at 1 year pi (Table 6).

**Table 6:** Post COVID-19 sequels that differentially reported at 28 days and 1 year after infection

Sequelae	Variables	Percentage^a^	χ^2 ^*p*-value	Odds ratio	95% CI	*p*-value
**Delta-infected group**						
Forgetfulness	Day 28	2.2%	0.001*	0.062	0.007–0.516	0.010*
	Year 1	26.5%				
Hair loss	Day 28	0.0%	<0.001*	-	-	-
	Year 1	38.2%				
**Omicron-infected group**						
Cough	Day 28	29.3%	0.021*	4.552	1.168–17.734	0.029*
	Year 1	8.3%				
Fatigue	Day 28	29.3%	0.007*	7.034	1.454–34.043	0.015*
	Year 1	5.6%				
**All participants**						
Forgetfulness at year 1	Delta	26.5%	0.044*	3.960	0.970–16.158	0.055
	Omicron	8.3%				
Hair loss at year 1	Delta	38.2%	0.003*	6.810	1.731–26.782	0.006*
	Omicron	8.3%				
	Male	10.3%	0.036*	0.249	0.064–0.972	0.045*
	Female	31.7%				
Headache at year 1	Delta	11.8%	0.034*	-	-	-
	Omicron	0.0%				
Joint pain at day 28	Age ≥ 60	12.9%	0.006*	-	-	-
	Age 18–59	0.0%				
Forgetfulness at day 28	Male	0.0%	0.025*	-	-	-
	Female	12.2%				
Joint pain at year 1	Male	0.0%	0.031*	-	-	-
	Female	14.6%				
**Adjusted odds ratio**						
Hair loss at year 1	Delta			8.678	2.055–36.652	0.003*
	Omicron					
	Male			0.181	0.042–0.783	0.022*
	Female					

a, Percentage of participants presented with the sequelae in each variable category; *, statistically significant at *p* ≤ 0.05

## Discussion

In this study, data and plasma samples were retrieved from COVID-19 patients admitted to three hospitals, one in Bangkok, Thailand, and two in nearby provinces sharing borders with Bangkok. The enrollment period was between July 2021 and August 2022, during which Thailand experienced epidemic waves caused by SARS-CoV-2 Delta (the fourth wave) and Omicron (the fifth wave) variants. B.1.617.2 (Delta) was the predominant strain during the fourth epidemic wave (July 2021–December 2021). B.1.1.529 (Omicron BA.1) was first detected in Thailand in December 2021. In January 2022, the clade B.1.1.529 (Omicron BA.2) emerged in Thailand and became the major variant by March 2022 [19]. There were substantial numbers of hospitalized patients and deaths at this time. At the beginning of the Delta wave, around 200,000 accumulated cases were reported. Cases rose to more than 2 million during the Delta wave and surpassed 4 million during the Omicron wave, reflecting the increased transmissibility of the Delta and Omicron variants compared to previous variants (Fig. 1). A longitudinal collection of serial plasma samples was performed in three hospitals for 1 year, although certain information, including clinical data, vaccination status, underlying diseases, and post-COVID-19 symptoms, was available only from Hospital A in Bangkok. Partial S gene sequencing was used to identify the SARS-CoV-2 variants from nasal swab specimens. Although the sequencing product covered only approximately 870 bp, corresponding to amino acid positions 10–301 on the S protein, this region contains mutations that can be used to distinguish between the Delta variant, Omicron BA.1, and Omicron BA.2, such as T19R and Δ156–157/R158G (specific to Delta), A67V and Δ143–145 (specific to Omicron BA.1), and L24S/Δ25–27 (specific to Omicron BA.2) [25]. The region also contains mutations specific to the Omicron XBB subvariant. While discrimination of additional variants may require the analysis of other regions, the sequencing of the partial S gene reported here was effectively used for the identification of Delta and Omicron variants.

Fever, cough, sore throat, congestion or runny nose, fatigue, and headache, which are common symptoms of respiratory infections, were generally observed in COVID-19 participants. However, while fever, cough, and fatigue were reported in comparable proportions among participants infected with the Delta and Omicron variants, sore throat, congestion or runny nose, and headache were more often associated with Omicron infections in this study. In a study from the UK that analyzed a large set of self-reported data from COVID-19 patients during the Delta and Omicron prevalence periods, sore throat was also more often associated with Omicron infections, whereas loss of smell, sneezing, runny nose, headache, fever, and hair loss were less often associated with Omicron than Delta infections [26]. A study from China comparing Omicron to Delta and Beta infections showed that participants infected by Omicron were more likely to have a sore throat, but the incidences of headache, diarrhea, taste loss, and anosmia were lower [27]. In our study, diarrhea, loss of taste (ageusia), and anosmia were reported as rare symptoms in both Delta and Omicron infections. Collectively, the studies suggest that, while other symptoms may vary across study populations, sore throat is strongly associated with the Omicron infection. Viral pneumonia was distinctively diagnosed more frequently in Delta-infected participants than Omicron-infected participants. This finding is in agreement with previous studies that reported reduced severity and hospitalization rates for Omicron infections compared to Delta infections [28, 29], despite Omicron’s higher transmissibility [30]. Ex vivo studies of explant cultures of human bronchi and lungs infected with different SARS-CoV-2 variants indicated that, despite Omicron replicating more efficiently than Delta and other variants in the bronchi, it replicated less efficiently in the lung tissues, which could explain its milder disease severity but higher transmissibility [31]. Omicron primarily localizes in the upper respiratory tract rather than penetrating deep into the lung tissue [30].

A higher proportion of patients infected with the Omicron variant had hypertension and dyslipidemia compared to those infected with the Delta variant. In one study, a higher proportion of patients infected with the Omicron variant had abnormally high total cholesterol and low-density lipoprotein levels compared to those infected with the Delta variant [28]. In contrast, they reported a higher frequency of hypertension in patients with the Delta variant than the Omicron variant. Hypertension was also reported to be more prevalent than other underlying conditions, such as obesity and diabetes mellitus, in COVID-19 patients [32]. We also found that underlying conditions, including hypertension, dyslipidemia, and diabetes mellitus, were associated with an age of ≥ 60 years. Although no significant association between age and infected variant was observed, the proportion of older participants (≥ 60 years old) was higher in the Omicron-infected group (43.9%) than the Delta-infected group (28.3%). A possible explanation for the higher prevalence of Omicron in older individuals and participants with underlying diseases may have been due to the relaxation of personal hygiene measures and social distancing after a prolonged period of living with the pandemic, particularly among individuals with comorbidities. Moreover, we found that diabetes was associated with disease severity, and asthma and hematologic disease were associated with shortness of breath. As revealed by previous studies, COVID-19 patients with diabetes had a higher chance of developing severe COVID-19 and increased mortality than those without diabetes [32, 33]. It has also been reported that smoking is associated with an increased risk of COVID-19 severity and mortality [34]. However, in this study, no association was found between smoking and disease severity. Although links between underlying conditions and specific SARS-CoV-2 variants could not be established, the overall findings emphasized that individuals with comorbidities require more attention when infected with respiratory viral diseases such as COVID-19.

Because of the vaccine implementation timeline, more than half of the Delta-infected participants were unvaccinated or had received only one dose of the vaccine, while all Omicron-infected participants were vaccinated with at least two doses. Vaccines available in Thailand require two doses, whether inactivated, viral vector-based, or mRNA vaccines. During the outbreak, various combinations of vaccines were used, as they were subject to availability (Fig. 4). This could have affected the antibody responses and complicated the evaluation of protective responses against SARS-CoV-2 in Thai individuals. We found that vaccination with fewer than two doses, regardless of the vaccine platform, was associated with increased disease severity, which may explain the higher proportion of pneumonia observed in Delta-infected participants than in those infected with Omicron. There was also evidence suggesting that, during the Omicron wave, unvaccinated individuals were more vulnerable to severe COVID-19 outcomes [35]. Although COVID-19 vaccines derived from the wild-type Wuhan strain cannot completely protect individuals from COVID-19 caused by the evolved variants, these vaccines have been proven effective in reducing the risk of severe disease, hospitalization, and death [36]. Vaccines can be important tools for fighting infectious diseases. Although complete protection remains a challenge, the benefits of vaccines in reducing disease severity are obvious. The development of multivalent and universal vaccines could be another strategy used to combat the disease.

Our group previously reported that IgA, total IgG, IgG1, and IgG3 are the major antibodies generated in response to the RBD of SARS-CoV-2 in Thai COVID-19 patients [22]. Therefore, to evaluate immune responses against Delta and Omicron variants in this study, IgG and IgA levels were determined using RBD-specific ELISA. Considering all participants, the IgA and IgG antibody levels generated against the Delta variant’s RBD peaked on day 14 and were maintained for at least 2 months. In the Delta-infected participants, antibodies against the Delta RBD were maintained for 1 year, but showed a waning trend. For Omicron-RBD antibodies, although a significant rise was observed in the convalescence phases, the antibody levels were lower and the pattern of antibody increase was not as clear as with the Delta-specific antibodies. A previous study revealed that IgA and IgG antibodies against the Delta and Omicron variants of SARS-CoV-2 significantly increased within 14 days and remained high for over 1 month. Both IgA and IgG antibodies showed similar trends, but the positivity rate for the IgG antibody was higher than that for IgA [37]. We observed ambiguous patterns of antibody responses in Omicron-infected participants, in which cross-reactive antibody levels against the Delta variant seemed to be higher, with a clear increasing trend, than those against the Omicron variant. An explanation for this may be prior infections with former variants causing immune imprinting [38]. However, the relatively low level and unclear pattern of specific antibodies to Omicron RBD detected in the Omicron group was in contrast to previous findings that showed a favorable neutralizing antibody response against Omicron variants in Omicron-infected patients [39]. Because ELISA was used for antibody detection in this study, we could not draw any conclusions on whether the detected antibodies were neutralizing antibodies or just binding antibodies; although in one study, neutralizing antibody and ELISA IgG antibody levels against SARS-CoV-2 were found to be correlated [40]. Regarding the rapid evolution of SARS-CoV-2 Omicron subvariants in the RBD region, this could affect the sensitivity of the ELISA assay. Molecular characterization revealed that BA.1 and BA.2 were detected in our samples, although the BA.2 recombinant RBD was used for ELISA plate coating, subject to availability. We found that BA.1 and BA.2 share approximately 97% amino acid sequence identity in the S1 subunit containing the RBD. How this difference affects the sensitivity and accuracy of the measurement requires further investigation.

Associations between antibody elevations and other factors were assessed in this study. Pneumonia was found to be associated with a rise in Delta-specific IgA and IgG in the Delta-infected participants. Several studies have shown that antibody responses were positively correlated with disease severity in COVID-19 patients [41–43]. Previous findings from Thailand revealed significantly higher IgG, IgA, and neutralizing antibody titers in COVID-19 patients with pneumonia than those without pneumonia [44]. Underlying diseases, including obesity, hypertension, and dyslipidemia, were found to be associated with a rise in cross-reactive Omicron antibodies in Delta-infected participants, whereas an age of ≥ 60 years was associated with a rise in cross-reactive Delta antibody levels in Omicron-infected participants. Antibodies against SARS-CoV-2 RBD in serum collected from an acute respiratory outpatient cohort were positively associated with age (over 45 years) and body mass index (BMI) [45], whereas a study in healthcare workers reported that central obesity and hypertension were associated with lower anti-SARS-CoV-2 antibody titers after vaccination with an mRNA vaccine [46]. Dyslipidemia was found to be linked to increased mortality and COVID-19 severity, with stronger associations observed in older, male, and hypertension patients [47]. We also found hypertension and diabetes mellitus to be associated with increased levels of Omicron-specific IgA in the Omicron-infected participants. An increase in serum IgA concentrations was reported as a generalized phenomenon among diabetic patients, especially in those with complications [48]. Obesity was found to be linked with decreased Delta-specific IgG at year 1. It was reported that the half-life of IgG decreased as BMI and body fat mass increased in rats and humans, possibly due to increased catabolism of IgG in obesity [49]. However, to gain an understanding of the variant-specific and cross-reactive antibody responses in COVID-19, which involve multiple factors, more samples and specific study designs are required, as findings from different study settings can gave different results.

Several post-COVID-19 symptoms were reported by the participants 28 days and 1 year pi. Cough and fatigue were generally reported by both Delta and Omicron participants on day 28 pi, and the symptoms seem to diminish over time. The decreases in cough and fatigue reported one year post-infection were more clearly seen in the Omicron group than in the Delta group. Among the Delta-infected participants, reports of forgetfulness and hair loss, which were rare on day 28, had increased 1 year after infection. Our results found that forgetfulness, hair loss, and headache at year 1 post-infection were significantly more frequently associated with the Delta infection than the Omicron infection. Although not statistically significant in this study, fatigue also seemed to be more closely associated with Delta infections in the long term. Previous studies have revealed the most prevalent long-COVID symptom to be fatigue, regardless of the SARS-CoV-2 variant involved in the infection [50, 51]. Fatigue, joint or muscle pains, and temporary hair shedding were found more often in Delta-infected patients than Omicron-infected patients post-infection [52]. Patients infected with the Omicron variant were associated with a lower risk of long-term sequelae than patients infected with previous variants [50, 53]. Female gender and the presence of various comorbidities were associated with an increased risk of developing persistent symptoms [54]. We also found in this study that females were significantly more likely to have symptoms such as forgetfulness, hair loss, and joint pain from 28 days up to 1-year post-infection. Hair loss has been reported as one of the most common post-COVID-19 recovery symptoms [55]. The association between COVID-19 infection and telogen effluvium, which is a common cause of diffuse non-scarring hair loss, appears stronger than that observed for other forms of hair loss. TE can occur 3–4 months after acute illness, use of certain medications, medical or surgical interventions, stress, high-grade fever, or nutritional deficiencies [55].

It is important to acknowledge the limitations of this study, which stemmed from the research being conducted during a pandemic emergency, when many factors could not be fully controlled. First, this study only included confirmed COVID-19 patients who were admitted to the hospitals. According to Thailand’s national guidelines, those with underlying conditions were given priority for admission. Consequently, some associations between demographic, clinical, and disease parameters, especially those related to disease severity and underlying conditions, may have been missed. Due to the small sample size and incomplete data from Hospitals B and C, most analyses and interpretations were based solely on data from Hospital A. However, Fig. 5 provides an overall picture of antibody responses across all sites. Given this limitation, the potential impact of population heterogeneity across different locations could not be addressed. Second, the sample and data collection spanned one year, resulting in some follow-up periods being missed. Quarantine measures after hospital discharge also made it impossible to follow up some patients, leading to gaps in their data. We cannot ensure that all participants experienced a first infection, as some might have had asymptomatic infections without realizing they were infected. During enrollment, we included hospitalized participants with current infections confirmed by real-time RT-PCR, but we did not address the issue of reinfection at the initial enrollment stage. However, during follow-up, we asked participants whether they had been reinfected after joining the study. Some participants reported reinfection after day 28, and their year 1 data were removed from the analysis. Because of incomplete data, statistical analyses were limited and multivariable analysis was challenging, so univariable analysis was performed instead. Lastly, information on the patients’ post-COVID-19 symptoms were collected via questionnaires, which may have introduced subjective bias, particularly for symptoms reported 1 year after infection.

## Conclusions

In this cross-sectional descriptive study, we analyzed associations among various parameters in SARS-CoV-2-infected Thai participants during the Delta and Omicron waves. Despite some common symptoms, different SARS-CoV-2 variants, such as Delta and Omicron, which emerged through viral evolution over a short period, exhibited some distinct characteristics. Viral factors and host factors (such as underlying diseases and vaccination status) are contributed to different disease outcomes. Vaccination and specialized healthcare for vulnerable individuals with comorbidities are crucial for appropriate management during pandemic surges. Currently, COVID-19 is no longer considered a global threat due to the availability of vaccines, herd immunity, and accumulated experience in disease management, all of which have led to reduced severity and hospitalization rates. Nevertheless, COVID-19 cases continue to be reported, driven by evolved strains of SARS-CoV-2. The knowledge gained from previously collected data remains vital for understanding the virus and disease characteristics, aiding preparedness for potential future threats from emerging SARS-CoV-2 variants or other novel viruses.

## Electronic supplementary material

Below is the link to the electronic supplementary material.

Supplementary Material 1

## Data Availability

Sequence data was deposited in the GenBank database, accession numbers PP411428 to PP411479. Questionnaire was provided as a supplementary file.

## Abbreviations

bp: Base pair
COVID-19: Coronavirus disease 2019
ELISA: Enzyme-linked immunosorbent assay
Ig: Immunoglobulin
OD: Optical density
pi: Post-infection
RBD: Receptor-binding domain
RT-PCR: Reverse transcription-polymerase chain reaction
S: Spike glycoprotein
SARS-CoV-2: Severe acute respiratory syndrome coronavirus 2
WHO: World Health Organization
