# Supplementary material for: Associations between clinical data, vaccination status, antibody responses, and post-COVID-19 symptoms in Thais infected with SARS-CoV-2 Delta and Omicron variants: a 1-year follow-up study
Source: BMC Infect Dis. 2024 Oct 7;24:1116. doi: 10.1186/s12879-024-09999-2 (PMC11460119; doi:10.1186/s12879-024-09999-2)
Supplement: Supplementary file 1 — Supplementary Material 1 [file 12879_2024_9999_MOESM1_ESM.pdf]

**Questionnaire – Analysis of immune responses and host susceptibility to COVID-19 infection (COVID-19 cases)**	**COVID-19 |__|__|__|**

**PATIENT INFORMATION**
**Enrollment Date**: |__I__| / |__I__|__| / |__I__|

○ answered by patient ○ answered by family ○ answered by other: _________________

Known source of COVID-19 cluster ○ Yes ○ No ○ Unknown

If yes, specify____

Have a history of returning from abroad after 2020 ○ Yes ○ No ○ Unknown
(If yes, from ____________________ to Thailand at the date |__I__| / |__I__|__| / |__I__|

Have a history of traveling outside the living area within 14 days before onset
○ Yes ○ No ○ Unknown

(If yes, travel to subdistrict:______________ province: ____________Date: |__I__| / |__I__|__| / |__I__|)

Have a history of living in COVID-19 hitting provinces in the past 1 month? ○ Yes ○ No ○ Unknown
(If yes, specify____)

Are you a healthcare worker at risk for COVID-19 infection? ○ Yes ○ No ○ Unknown
If yes, specify detail of work____

COVID-19 vaccination ○ Yes ○ No ○ Unknown
If yes, number of doses received: ○ 1 ○ 2 ○ Unknown

Product name of COVID-19 vaccine_________________

Date of vaccine dose 1: |__I__| / |__I__|__| / |__I__| Date of vaccine dose 2: |__I__| / |__I__|__| / |__I__|

Source of information ○ Documented evidence ○ Recall

completed by __________________ **Date**: |__I__| / |__I__|__| / |__I__| **Time**: |__I__|:|__I__| (24:00)

APPROVED
ETHICS COMMITTEE
FACULTY OF TROPICAL MEDICINE
MAHIDOL UNIVERSITY

Digitally signed by Faculty of Tropical Medicine, Mahidol University
DN: c=TH, st=Bangkok, l=Ratchathewi, o=Faculty of Tropical Medicine, Mahidol University, cn=Faculty of Tropical Medicine, Mahidol University, email=tmectropmed@mahidol.ac.th
Date: 2021.06.14 08:31:34 +07'00'

Questionnaire – Analysis of immune responses and host susceptibility to COVID-19 infection (COVID-19 cases)	COVID-19 |__|__|__|

PATIENT INFORMATION

28 DAY VISIT AFTER ENROLLMENT

Date |__I__| / |__I__|__| / |__I__| ○ **In person** ○ **By phone**

○ **Unable to contact or determine status** ○ **Died; date of death |__I__| / |__I__|__| / |__I__|**

○ answered by patient ○ answered by family ○ answered by other: _________________

COVID-19 vaccination ○ Yes ○ No ○ Unknown
If yes, number of doses received: ○ 1 ○ 2 ○ Unknown

Product name of COVID-19 vaccine_________________

Date of vaccine dose 1: |__I__| / |__I__|__| / |__I__| Date of vaccine dose 2: |__I__| / |__I__|__| / |__I__|

Source of information ○ Documented evidence ○ Recall

Are you taking any medications now? ○ Yes ○ No **If yes, list medications:**

1.	2.
3.	4.

Since you left the hospital, in general do you feel: ○ improved ○ the same ○ worse

**Since you left the hospital, have you had any of the following symptoms?**			○ Yes ○ No ○ Unknown
○ Fatigue<br>○ Cough<br>○ Fever<br>○ Sore throat<br>○ Congestion or runny nose<br>○ Shortness of breath<br>○ Headache<br>○ Nausea or vomiting	○ Constipation<br>○ Diarrhea<br>○ Weight loss<br>○Skin rash<br>○ Hair loss	○ Forgetfulness<br>○ Difficulty with thinking and concentration<br>○ Depression<br>○ Fast-beating or pounding heart (heart palpitations)	○ Chest pain<br>○ Joint pain<br>○ Muscle pain<br>○ pain at other site(s):_____
○ other, describe: ____			

Have you been *diagnosed* with any new medical problems? ○ Yes ○ No **If yes, list problems:**

____

Have you been re-admitted to hospital for any reason? ○ Yes ○ No **If yes, list reason:**

____

Date of re-admission |__I__| / |__I__|__| / |__I__| **Hospital** ________________________________

Other information, specify____

completed by __________________ **Date**: |__I__| / |__I__|__| / |__I__| **Time**: |__I__|:|__I__| (24:00)

APPROVED
ETHICS COMMITTEE
FACULTY OF TROPICAL MEDICINE
MAHIDOL UNIVERSITY

Questionnaire – Analysis of immune responses and host susceptibility to COVID-19 infection (COVID-19 cases)	COVID-19 |__|__|__|

PATIENT INFORMATION

12 MONTH VISIT AFTER ENROLLMENT

Date |__I__| / |__I__|__| / |__I__| ○ **In person** ○ **By phone**

○ **Unable to contact or determine status** ○ **Died; date of death |__I__| / |__I__|__| / |__I__|**

○ answered by patient ○ answered by family ○ answered by other: ________________

COVID-19 vaccination ○ Yes ○ No ○ Unknown
If yes, number of doses received: ○ 1 ○ 2 ○ Unknown

Product name of COVID-19 vaccine________________

Date of vaccine dose 1: |__I__| / |__I__|__| / |__I__| Date of vaccine dose 2: |__I__| / |__I__|__| / |__I__|

Source of information ○ Documented evidence ○ Recall

Are you taking any medications now? ○ Yes ○ No **If yes, list medications:**

1.	2.
3.	4.

Since you left the hospital, in general do you feel: ○ improved ○ the same ○ worse

**Since you left the hospital, have you had any of the following symptoms?**			○ Yes ○ No ○ Unknown
○ Fatigue<br>○ Cough<br>○ Fever<br>○ Sore throat<br>○ Congestion or runny nose<br>○ Shortness of breath<br>○ Headache<br>○ Nausea or vomiting	○ Constipation<br>○ Diarrhea<br>○ Weight loss<br>○Skin rash<br>○ Hair loss	○ Forgetfulness<br>○ Difficulty with thinking and concentration<br>○ Depression<br>○ Fast-beating or pounding heart (heart palpitations)	○ Chest pain<br>○ Joint pain<br>○ Muscle pain<br>○ pain at other site(s):_____
○ other, describe: ____			

Have you been *diagnosed* with any new medical problems? ○ Yes ○ No **If yes, list problems:**

____

Have you been re-admitted to hospital for any reason? ○ Yes ○ No **If yes, list reason:**

____

Date of re-admission |__I__| / |__I__|__| / |__I__| Hospital ______________________________

Other information, specify____

completed by ________________ **Date**: |__I__| / |__I__|__| / |__I__| **Time**: |__I__|:|__I__| (24:00)

APPROVED
ETHICS COMMITTEE
FACULTY OF TROPICAL MEDICINE
MAHIDOL UNIVERSITY
